# Statins in the critically ill

**DOI:** 10.1186/2110-5820-2-19

**Published:** 2012-06-18

**Authors:** Isabelle De Loecker, Jean-Charles Preiser

**Affiliations:** 1 Department of Intensive Care, Erasme University Hospital, Route de Lennik 808, B-1070, Brussels, Belgium

**Keywords:** Mevalonate, HMG-CoA reductase, Sepsis, Acute lung injury, Acute respiratory distress syndrome, Subarachnoid hemorrhage

## Abstract

The use or misuse of statins in critically ill patients recently attracted the attention of intensive care clinicians. Indeed, statins are probably the most common chronic treatment before critical illness and some recent experimental and clinical data demonstrated their beneficial effects during sepsis, acute lung injury (ALI)/acute respiratory distress syndrome (ARDS), or after aneurismal subarachnoidal hemorrhage (aSAH). Due to the heterogeneity of current studies and the lack of well-designed prospective studies, definitive conclusions for systematic and large-scale utilization in intensive care units cannot be drawn from the published evidence. Furthermore, the extent of statins side effects in critically ill patients is still unknown. For the intensive care clinician, it is a matter of individually identifying the patient who can benefit from this therapy according to the current literature. The purpose of this review is to describe the mechanisms of actions of statins and to synthesize the clinical data that underline the relevant effects of statins in the particular setting of critical care, in an attempt to guide the clinician through his daily practice.

## Review

### Background

The use or misuse of statins in critically ill patients recently attracted the attention of intensive care clinicians for several different reasons. First, statins are probably the most common chronic treatment before critical illness. These lipid-lowering drugs are widely prescribed (18 million prescription purchase in France and 173.7 million in the United States) [1,2], because they improve survival in patients with cardiovascular disease [3,4] and in apparently healthy persons without hyperlipidemia but with elevated high-sensitivity C-reactive protein levels [5]. Second, the adverse effects of statins, including liver test abnormalities and rises in the plasma levels of creatine kinase, explain the withholding of statin treatment during the stay in intensive care units. Third, the continuation or discontinuation of a previous statin therapy use during hospitalization could be associated with specific effects. The importance of a prior statin treatment must be placed in the context of the paper by Heeschen et al. on the risk of statin removal in patients with acute arteriosclerotic cardiovascular disease [6]. Heeschen et al. investigated the effects of statins on the cardiac event rate in 1,616 patients of the Platelet Receptor Inhibition in Ischemic Syndrome Management (PRISM) study who had coronary artery disease and chest pain during the previous 24 h. If the statin therapy was withdrawn after admission for acute coronary syndrome, cardiac risk increased compared with patients who continued statins (2.93 [95% confidence interval (CI), 1.64–6.27]; *P* = 0.005) and tended to be higher compared with patients who never received statins (1.69 [95% CI, 0.92–3.56]; *P* = 0.15). This was related to an increased event rate during the first week after onset of symptoms and was independent of cholesterol levels. In a multivariate model, continuation of statin therapy (*P* = 0.008) was an independent predictor of favorable outcome. Fourth, some experimental and clinical data demonstrated beneficial effects of statins during sepsis, acute lung injury (ALI)/acute respiratory distress syndrome (ARDS), or after subarachnoidal hemorrhage (SAH) in relation with the so-called pleiotropic effects of this class of drugs [7]. These effects could benefit to these patients in relation with the associated anti-inflammatory, immunomodulatory, antithrombotic, and antioxidant properties found independently of the lipid-lowering properties. Because statins do not target individual inflammatory mediators, they could modulate the overall magnitude of the inflammatory response [8]. A recent study reported that preadmission use of statins was associated with a reduction in 30-day and 1-year mortality of a cohort of 12,483 critically ill patients [9]. Even patients under statin treatment developing multiple organ dysfunction syndrome (MODS) seems to have a better outcome than age- and sex-matched MODS patients without statin therapy [10].

These impressive findings underline the need for a review of the mechanisms of action and clinically relevant effects of statins in the particular setting of critical care. Therefore, we reviewed the literature retrieved on PubMed, using the following key terms: statins; hydroxymethylglutaryl-CoA reductase inhibitors; intensive care; ICU; critical care; sepsis; infection; ALI; ARDS; stroke; subarachnoid hemorrhage.

### Mechanisms of action of statins

**Figure 1:**
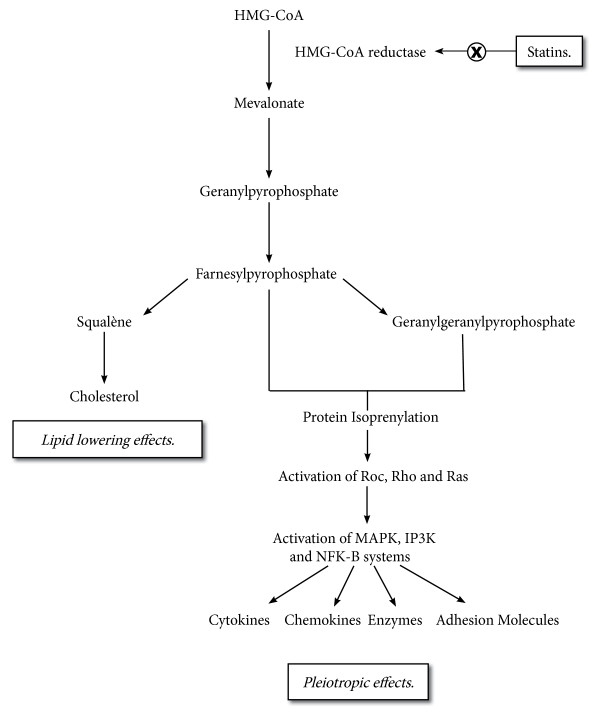
**Effects of statins on the cholesterol biosynthesis pathway.** HMG-CoA reductase inhibition by statins reduces intracellular mevalonate levels. Consequently, not only cholesterol levels are reduced but also the intermediary products farnesylpyrophosphate and geranylgeranylpyrophosphate. These latter two provide binding sites for protein isoprenylation, and activation.

Figure 1 displays schematically the mechanisms of action of statins. Intracellularly, statins inhibit the 3-hydroxy-3-methylglutaryl-CoA reductase (HMG-CoA reductase), thereby decreasing the synthesis of cholesterol. Besides this well-known effect, the inhibition of mevalonate synthesis by statins also results in the decrease of intermediary products of this cascade, including farnesylpyrophosphate and geranylgeranylpyrophosphate. These molecules are involved in the activation by isoprenylation of small GTP-binding proteins (Roc, Rho, and Ras). This event triggers the activation of transcription factors, which are involved in the pleiotropic effects of statins. The functional consequences of these actions are numerous and include anti-inflammatory and anti-oxidant effects, immune modulation, antithrombotic effects, protection of the endothelial function, and activation of vitamin D.

### Anti-inflammatory and immunomodulatory effects

**Figure 2:**
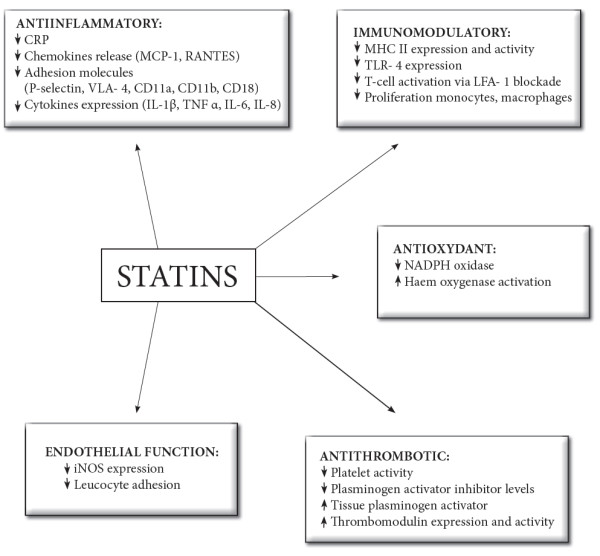
**Pleiotropic effects of statins.** Statins have anti-inflammatory, immunomodulatory, antithrombotic, and antioxidant properties found independently of their lipid-lowering properties. Because statins do not target individual inflammatory mediators, they could modulate the overall magnitude of the inflammatory response.

The inhibition of mevalonate synthesis by statins results in a lesser activation of the small GTP-binding proteins, which play a key role in the activation (molecular on/off switches) of intracellular inflammatory signalling pathways. Consequently, the response of the inflammatory intracellular signalling pathway upon stimulation is reduced, although not fully abolished. In particular, the activation of nuclear factor kappa B, mitogen-activated protein kinase (MAPK), and phosphatidylinositol-3 kinase (PI3K) systems by isoprenylated protein kinases is blunted (Figure 1). Therefore, the expression of cytokines, acute phase proteins, chemokines, adhesion molecules, and enzymes is partially inhibited in the presence of statins (Figure 2) [11]. By their action on chemokines and adhesion molecules, statins directly influence leucocyte function by a direct inhibition of the major histocompatibility complex type II (MHC II) gene of antigen presenter cells [12] and an allosteric block site of the lymphocyte function-associated antigen (LFA-1) [13,14], which has a significant role in lymphocyte adhesion and activation. Statins also decrease the expression of Toll-like receptor 4 (TLR4) [15-17], possibly by an alteration in cholesterol-rich membrane domains as observed in brain plasma membranes [18]. The clinical consequences of these anti-inflammatory and immunomodulatory effects will be discussed later in this review. 

#### *Vascular effects: Antithrombotic effects and preservation of endothelial function*

The cardiovascular effects of statins were the major focus of clinical research, because the beneficial effects of statin therapy were reported in patients with cardiovascular diseases. Besides the prevention of atherosclerosis by their lipid-lowering properties, statins also modulate coagulation and exert antithrombotic effects via a number of mechanisms (Figure 2), including a decrease in the activity of platelets, an increase in the tissue plasminogen activator and a decrease of its inhibitor, and an enhancement in the expression and functional activity of thrombomodulin, an essential cofactor for protein C activation [19]. Statins might thus enhance fibrinolysis by its effect of protein C.

Endothelial function also is preserved by statins, in relation with the maintenance of endothelial constitutive nitric oxide synthase (NOS) activity. On the other hand, excessive vasodilatation, loss of systemic vascular resistance, and vascular leak could be prevented by the inhibition of the inducible isoform of NOS [20-22].

#### *Antioxidant*

Oxidative stress is defined by an imbalance between increased levels of reactive oxygen species (ROS) and a low activity of antioxidant mechanisms. An increased oxidative stress can induce damage to the cellular structure and potentially destroy tissues. Therefore, the prevention of oxidative stress is probably desirable in critically ill patients to minimize the risk of new organ failures [23-26]. Statins exert antioxidant effects by the inhibition of the NADPH oxidase [27]. In case of cellular hypoxia and reoxygenation, the activation of heme oxygenase by statins could represent a protective mechanism against oxidative stress-related cellular damage [28-31].

#### *Vitamin D*

Cells of the innate and adaptative immune system express the vitamin D receptor and respond to stimulation by 1,25-dihydroxyvitamin D [1,25(OH)2D] [32], the active form of vitamin D. Moreover, 1,25(OH)2D leads to enhanced expression of human cathelicidin, which is an endogenous antimicrobial peptide active against a broad spectrum of infectious agents [33].

Statin therapy increases vitamin D major circulating form, 25-hydroxyvitamin D [25(OH)D] [34,35], by a mechanism that is still unclear, but could have beneficial effects on infection control [36,37].

#### *Comparison with corticosteroids*

Table 1 compare the anti-inflammatory, immunomodulatory, antioxidant, and endothelial effects of statins and corticosteroids. Besides some minor differences in their respective effects, the major distinction lies into the time to onset of action. On the opposite of corticosteroids of which antiinflammatory effects can be seen after less than 24 hours of treatment, statins only achieve a measurable anti-inflammatory effect after 7–14 days of treatment [38,39]. This delayed effect seems to make them an unlikely candidate for suppressing systemic inflammation in the acute phase of sepsis, as documented by two recent studies that found no reduction in markers of inflammation [40,41]. 

**Table 1:** Statins and corticosteroids

**Effects**	**Statins**	**Corticosteroids**
**Anti-inflammatory**	↓ CRP↓ Chemokine release (MCP-1, RANTES)↓ Cytokines (IL-1β, TNF α, IL-6, IL-8)↓ Adhesion molecule (P-selectin, VLA 4, CD11a, CD11b, CD18)	↓ CRP↓ Chemokine release (MCP-1, RANTES)↓ Cytokines (IL-1β, TNF α, IL-6, IL-8) but ↑ IL-10, one of the major anti-inflammatory cytokines↓ Adhesion molecule (ICAM-1, ELAM-1)↑Production of anti-inflammatory monocyte subtype.
**Immunomodulatory**	↓ MCH II expression and activity.↓ TLR-4 expression.↓↓ Proliferation of monocytes and macrophages.↓ Lymphocytes T activation	↑ MCH II expression on monocytes and eosinophiles.↓ CD 14 expression on monocytes↓ Complement activation.↓ Proliferation of lymphocytes.↓ Neutrophils aggregation and chemotactism.↑ Apoptosis of mature lympho T, eosinophils, endothelial cells and precursors of dendritic cells, but ↓ apoptosis of neutrophils.↑ Phagocytosis of apoptic neutrophils.
**Endothelial function**	↓ iNos expression↓ Leucocyte adhesion	↓ iNos expression↓ Leucocyte adhesion↓ Synthesis of COX-2, PGE-1 and prostacyclin↓ Production of Vascular endothelial growth factor↑ Sensitivity to α-agonist
**Antioxidant**	↓ NADPH oxidase↑ Haem oxygenase	↓ NADPH oxidase↓ release of superoxide from neutrophils
**Antithrombotic**	↓Platelet activation↓Plasminogen activator inhibitor levels↑Tissue plasminogen activator↑Thrombomodulin expression and activity	↓Platelet activation and aggregation.

Comparison between the anti-inflammatory, immunomodulatory, endothelial, antioxidant, and antithrombotic effects of statins and corticosteroids.

### Effects of statin therapy during critical illness

Although possible effects of statins might influence the outcome of patients with other conditions, documented effects were reported during sepsis, ALI/ARDS, and after SAH.

#### *Sepsis*

Sepsis is characterized by systemic inflammation and dysregulation of the coagulation cascade and remains a major source of morbidity and mortality in ICUs [42-45]. The currently available evidence suggests that the pleiotropic effects of statins could be beneficial during sepsis. The first encouraging clinical observations of improved outcome in septic patients receiving statins fostered the conduct of research in experimental models of sepsis.

#### *Experimental studies*

Two studies on animal models evaluated the effect on mortality of statins given twice before the induction of sepsis. Both studies demonstrated an improved survival in animals treated with statins. Ando et al. [46] showed that cerivastatin pretreatment of mice at 12 and 1 hour before lipopolysaccharide (LPS)-induced sepsis improved the rate of 7-day survival from 26.7% in nonpretreated group to 73.3% in cerivastatin-pretreated group. Merx et al. [47] evaluated the effects of statins given at 18 and 3 hours before polymicrobial sepsis using a rodent model of caecal ligation and puncture (CLP) and found a median survival time extended to 108 hours from 28 hours in untreated mice. This improvement in the survival rate of simvastatin-treated mice was associated with a complete preservation of cardiac function at 20 hours and a preserved responsiveness to dobutamine, in contrast to the untreated group, whose cardiac function and responsiveness to dobutamine were severely impaired.

Another rat study of endotoxic shock [21] confirmed an enhanced vascular responsiveness after simvastatin pretreatment 20 minutes before LPS. The impaired pressor response to phenylephrine was preserved compared with rats treated with untreated animals.

Only one animal study evaluated the effect of various statins after the onset of sepsis [48]. Statin treatment was initiated 6 hours after CLP, when profound hemodynamic alterations were present. Survival time was only 23 ± 1.2 hours in the untreated group, whereas it was extended to 39 ± 3.9 hours after treatment with atorvastatin, to 40 ± 4.2 hours after pravastatin, and to 37 ± 3.6 hours after simvastatin (all *P* < 0.05 versus untreated). No change in the survival time was observed after fluvastatin. Cardiac output measured at 20 hours after sepsis induction remained stable in CLP mice treated with atorvastatin, pravastatin, or simvastatin after sepsis induction (*P* = NS vs. preoperative), whereas it decreased significantly in placebo or fluvastatin-treated mice. The responsiveness to catecholamines, including dobutamine was restored in CLP mice by treatment with atorvastatin, pravastatin, or simvastatin.

### Clinical data

#### *Cohort studies*

A meta-analysis summarizing the effects of statins on mortality in patients with infection and/or sepsis [49] seems to support the hypothesis of a protective effect of statins during sepsis.

The analysis included 20 studies (18 cohort studies (12 retrospective and 6 prospective), 1 matched cohort study, and 1 randomized control trial). Of those 20 studies, 15 showed a decreased mortality in patients receiving statins with adjusted odds ratios (ORs) ranging from 0.06 to 0.75 [50-64]. Four studies showed a trend toward a benefit from statins although not reaching statistical significance [65-68]. Only one study reported an increased mortality in patients taking statins [69].

The primary meta-analysis regarding mortality from any cause at different time, according to 20 studies that pooled 265,558 patients, demonstrated a significant protective effect from statins in patients with infection and/or sepsis compared with no statin treatment or placebo (OR, 0.49; 95% CI, 0.37–0.61) [50-69].

This meta-analysis supports the hypothesis of a protective effect of statins during sepsis [49]. However, besides limitations regarding the heterogeneity and design of the analyzed studies and exposure definition, the handling of potential sources of bias and confounding, such as the healthy user effect or indication bias, limits the interpretation of these data. A recent prospective cohort study, specifically designed to avoid those bias, found no evidence of a protective effect for statin use on clinical outcomes [40].

This study enrolled 1,895 subjects hospitalized with community-acquired pneumonia. It first compares subjects with prior statin use with those with no prior use and then compares continued in-hospital therapy with no prior use or no in-hospital use. There were no difference in severe sepsis risk between statin users and nonusers for prior (30.8% vs. 30.7%, *P* = 0.98) or continued statin use (30.2% vs. 30.8%, *P* = 0.85) in univariate analyses and after adjusting for patient characteristics and propensity for statin use. Interestingly, users whose statin was discontinued in the hospital had nearly twice the mortality of those whose statin was continued (15.3% vs. 7.9%, *P* = 0.048). After accounting for likelihood of statin use by including a propensity score in each model, there was no detectable benefit of either prior (adjusted OR, 0.9; 95% CI, 0.63–1.29; *P* = 0.57) or continued statin use (adjusted OR, 0.73; 95% CI, 0.47–1.13; *P* = 0.15).

First, in this study statin users were universally more likely to have healthy user indicators (being insured, living at home, being of good functional status, receiving vaccinations, taking daily aspirin, and quitting smoking) that can positively influence mortality in CAP. As these statin users’ characteristics are supported by other work in this area [70-72], this healthy-user effect may account for better outcomes shown in previous studies [50-64].

Second, this analysis refutes prior studies that support a protective effect of in-hospital ongoing statin therapy probably due to indication bias. Indeed, the decision to continue statin in the hospital depends on how sick the patient is and whether they are able to take medications by mouth. In this study, the inclusion of propensity for continued statin use in the mortality models universally moved the adjusted OR closer to unity and the *p* value toward greater degrees of insignificance, suggesting no association between 90-day mortality and cessation of statin therapy.

These findings seem to be confirmed by a randomized, placebo-controlled trial that does not support a beneficial role of continuing preexisting statin therapy on sepsis and inflammatory parameters [41]. This small trial tested the hypothesis that continuation of therapy with statins influences the inflammatory response to infection and that cessation may cause an inflammatory rebound. One hundred and fifty patients on preexisting statin therapy requiring hospitalization for infection were randomized to receive atorvastatin (20 mg) or matched placebo. The primary end point was progression of sepsis during hospitalization. The rate of decline of severe sepsis was similar between the groups (odds ratio, 1.17 [0.56-2.47], *P* = 0.7 day 3; 0.85 [0.21-3.34], *P* = 0.8 day 14) and IL-6 and C-reactive protein declined in both groups with no statistically significant difference (*P* = 0.7 and *P* = 0.2, respectively). Hospital mortality was 6.6%, with no difference between the groups (6 [8%] of 75 statin group; 4 [5.3%] of 75 placebo group; not significant).

These observational trials studied the effects of chronic treatment before infection. The effect of statin treatment initiated just before the onset of sepsis a patients at-risk population or even after the onset of sepsis were not assessed. Obviously, prospective, randomized trials over acute statin therapy are needed.

#### *Placebo-controlled, randomized trial*

Until now, only one double-blind, placebo-controlled, randomized trial has tried to determine whether acute statin therapy reduces the incidence of severe sepsis in patients with acute bacterial infection [73]. Unfortunately, because of very slow recruitment rate, this study was stopped prematurely after 10 months and enrolled only 83 subjects compared with the 1,080 planned in an estimated 24-month period. Several other clinical trials are now underway to examine the potential clinical benefit of statins in sepsis.

#### *Ongoing clinical trials*

Simvastatin and severe sepsis trial (SIMSEPT) will be the first double-blind, randomized, placebo-controlled trial of simvastatin (40 mg) in the treatment of severe sepsis in humans. It will investigate the effect of simvastatin on important inflammatory markers and monitor the safety and feasibility of administering simvastatin to patients with severe sepsis [74]. The STATInS trial is a phase II, randomised, placebo-controlled study of the safety, pharmacokinetics, and effect on inflammatory marker levels of atorvastatin in intensive care patients with severe sepsis [75]. Two other phase II trials are evaluating simvastatin in adults hospitalized with sepsis with the primary outcome being time to clinical stability in one trial [76] and time to shock reversal in the other [77].

### ALI/ARDS

ARDS is a disease of multifactorial etiology characterized by aspecific morphologic lesion termed “diffuse alveolar damage” (DAD) [78]. Current understanding places dysregulated systemic inflammation—with persistent elevation of circulating inflammatory and hemostasis markers over time—as the central pathogenetic process for dysfunction and failure of vital organs, the leading cause of (short- and long-term) morbidity and mortality in patients with ARDS [79].

Even with the use of protective ventilation [80], the damaged lung is still susceptible to ventilation-induced injuries [81]. There is, therefore, an urgent need for other strategies to improve clinical outcome in ALI/ARDS [82]. Several observational studies suggested that statins could represent a useful therapeutic adjunctive modality, as a benefit of prior statin use was found in patients with pneumonia [51,59,63].

#### *Experimental studies*

Animal studies have demonstrated that statins attenuate lung injury in models, such as ischemia-reperfusion, peritonitis, and aerosolized LPS [83-86]. In thirty healthy volunteers, a double-blind study compared the effects of a pretreatment with simvastatin 4 days before LPS inhalation to placebo [87]. A lower neutrophil count (3 × 10^5^/ml [1.8-8.1] vs. 8.5 × 10^5^/ml [4.4-16.2]; *P* = 0.05 vs. placebo) and activity in the alveolar space, partially due to an increase in neutrophil apoptosis was found in the bronchiolo-alveolar lavage fluid (BALF) of the subjects randomized to simvastatin. The increase in alveolar neutrophil apoptosis may be related to a reduced pulmonary and systemic inflammation after LPS inhalation compared with placebo. Interestingly, the simvastatin pretreated group also demonstrated a reduction in MMPs and tumor necrosis factor α concentrations in the BALF. The decrease in MMP-7 (*P* = 0.03 vs. placebo) could be particularly relevant, because this factor is required for neutrophil influx during ALI [88].

### Clinical data

#### *Cohort studies*

Most observational studies show a benefit of prior statin use in patients with pneumonia, suggesting a potential modulation in pulmonary inflammation [51,54,59,61-63,89-91]. One observational [92] and one retrospective [93] cohort study of patients with ALI/ARDS reported a trend toward reduced mortality in patients who were receiving statins before hospitalization.

#### *Placebo-controlled, randomized trials*

Recently, Craig et al. [94] reported a randomized, double-blinded, placebo-controlled trial in 60 patients with ALI. Patients were receiving 80 mg of simvastatin or placebo until cessation of mechanical ventilation or up to 14 days. Although the pulmonary anti-inflammatory effect of simvastatin was confirmed early in the course of treatment (2.5- to 3-fold reductions in BALF IL-8 and IL-6 concentrations compared with placebo), there was no difference in extravascular lung water index at day 7 (13.7 [7.1] vs. 13.4 [8.0] for placebo; *P* = 0.9). Nonetheless, at day 14 the simvastatin-treated group tended to achieve improved oxygenation index (*P* = 0.08), reduction in plateau pressure (*P* = 0.09), and better lung injury score (*P* = 0.12). Very interestingly, nonpulmonary organ failures were favorably influenced by simvastatin, as the coagulation, renal, and cardiovascular components of the SOFA score were all significantly improved at day 14 in the simvastatin group. The preservation of endothelial function by statins was suggested to account for the improvement in nonpulmonary organ dysfunction. Interestingly, this study also shows that, despite the precocity of the anti-inflammatory effects, a prolonged treatment may be required for these effects to be translated into improvements in lung function. If this study has shown that simvastatin has an effect on systemic and, to a lesser degree, pulmonary outcomes in patients with ALI, it was not powered to detect a difference in duration of ventilation or mortality. Table 2 compares the effects of statins and low-dose corticosteroids versus placebo on the ventilatory status and outcome of patients with ARDS [95,96]. 

**Table 2:** Effects of statins and low-dose corticosteroids versus placebo on the ventilatory status and outcome of patients with ARDS

	**Statins (80 mg/day)**			**Low-dose corticosteroids (1 mg/kg/day)**					
**Variables**	**Simvastatin^95^(n = 30)**	**Placebo(n = 30)**	***P***	**Methylprednisolone^96^ (n = 63)**	**Placebo(n = 28)**	***P***	**Methylprednisolone^97^ (n = 55)**	**Placebo(n = 24)**	***P***
PaO_2_/FiO_2_ day 7	199 ± 76	199 ± 76	NS	256 ± 19	179 ± 21	0.006			
LIS day 7	2 ± 0.78	2.1 ± 0.7	NS	2.14 ± 0.12	2.68 ± 0.14	0.004			
Ventilator free days	8.2 ± 8.1	9.1 ± 8.7	NS	16.5 ± 10.1	8.7 ± 10.2	0.001			
Duration of ventilation	18.6 ± 14.6	18.6 ± 14.6	NS	5 (3-8)	9.5 (6-19.5)	0.002	5 (0-64)	9.5 (0-63)	0.005
ICU stay				7 (6-12)	14.5 (7-20.5)	0.007			
ICU survival (%)	21 (70)	21 (70)	NS	50 (79.4)	16 (57.4)	0.03	44 (80)	14 (58)	0.05
Hospital stay	51.2 ± 39.3	48 ± 37.4	NS	13 (8-21)	20.5 (10.5-40.5)	0.09			
Hospital survival (%)	19 (63)	19 (63)	NS	48 (76.2)	16 (57.1)	0.07			

Data are presented as mean ± SD, no. (%), and median (interquartile range).

*NS* not significant.

#### *Ongoing clinical trials*

The National Institutes of Health Acute Respiratory Distress Syndrome Network is currently recruiting patients with sepsis-induced ALI into the Statins for Acutely Injured Lungs study (SAILS) [97], which is powered for clinical outcomes measures with an estimated enrollment of 1,000 patients and a completion date estimated for September 2012.

### Statins and aneurismal subarachnoid hemorrhage (aSAH)

Vasospasm and related delayed ischemic neurologic deficits (DINDs) remain a major cause of morbidity and mortality after aneurysmal subarachnoid hemorrhage (aSAH). Current medical treatment options are limited to triple-H therapy (hypertensive, hypervolemic, hemodilution) [98] and calcium-channel blocker nimodipine [99]. Potentially, statins might exert beneficial effects in aSAH, as a result of the protection of endothelial function by an increased expression of endothelial NOS, by the promotion of angiogenesis, by the prevention of excessive platelet activation, or via their antioxidant or anti-inflammatory effects. Regardless of the underlying mechanism, in the presence of statins the diameter of the vasospastic vessels are increased, endothelial function is improved and most importantly, ischemia is prevented [100].

### Clinical data

#### *Meta-analyses of placebo-controlled, randomized trials*

In 2008, a meta-analysis of three studies concluded that in patients with aSAH, statins reduced the incidence of vasospasm, DINDs, and mortality [101] However, this meta-analysis was criticized for its methodology and interpretation of results [102,103]. Recently, a meta-analysis of four randomized [64,104-108], double-blinded, placebo-controlled trials investigating the effects of acute statin treatment on vasospasm, DINDs, and outcome was performed, including 190 statin-naïve patients with aSAH. For the analysis of the occurrence of transcranial Doppler (TCD) vasospasm, data from three of the four studies were available [106-108]. The overall number of patients who had TCD vasospasm was 42 in the statin group and 46 in the placebo group (pooled relative risk (RR), 0.99; 95% CI, 0.66–1.48). The total number of patients who developed DINDs was 20 in the statin group and 39 in the placebo group, but this difference was not statistically significant (pooled RR, 0.57; 95% CI, 0.29–1.13). However, a subgroup analysis for the type of statins showed that patients randomized to pravastatin (n = 40) had a significant lower risk of DINDs (RR, 0.17; 95% CI, 0.04–0.7) compared with placebo.

As for TCD vasospasm and DINDs, definitions used for poor outcome were different in each study. In two studies, functional outcome was assessed with the modified Ranking Scale (mRS) [106,107]. The Glasgow Outcome Scale scores of the latter study were transformed to a Rankin grading [108]. Furthermore, the timing of measuring differed between groups either at discharge or at 6 months. The overall number patients with poor outcome was 38 in the statin group and 42 in the placebo group (pooled RR, 0.92; 95% CI, 0.68–1.24). The results of this meta-analysis do not lend statistically significant support to a beneficial effect of statins in patients with aSAH.

Another meta-analysis of six RCTs concluded that statins reduced the occurrence of DINDs (OR, 0.38 (0.23-0.65); *P* < 0.001) [106-112]. However, this analysis included two RCTs in which patients were “pseudo-randomized,” i.e., unblended or not placebo-controlled [110,111]. When these two latter studies were omitted, there was a larger heterogeneity in the effects of statins on DINDs (*I*^*2*^ *=* 47%).

#### *Cohort studies*

If RCTs are clearly the preferred study design to assess treatment efficacy, the small number of patients in these RCTs may unbalance the baseline characteristics and, thus, influence the results. The addition of observational studies can increase greatly the sample size, thereby reducing the chance of type II errors.

In the same paper, Kramer analyzed six observational studies (five cohort, one case–control), including 1,542 patients of whom 385 received statins [109,112-117]. Statin-use was not associated with any reduction in DINDs (OR, 0.96 (0.71–1.31); *P* = 0.8), mortality (OR 1.16 (0.78–1.73); *P* = 0.47) or poor neurological recovery (OR, 1.2 (0.84–1.72; *P* = 0.31). When the results of these six observational studies were combined with those of the six RCTs, statins had no statistically significant effect.

Interpreting results about possible beneficial effects of immediate statins therapy in statin-naïve patients following aSAH is limited by small sample sizes and low to moderate quality of available RCTs and very low quality for observational studies. Some of the inconsistencies of results may reflect substantial differences among studies design.

#### *Ongoing clinical trials*

Four, registered, clinical trials are currently assessing the efficacy of statins aSAH. The SimvaSTatin in Aneurysmal Subarachnoid Haemorrhage (STASH) trial [118] is a multicenter, placebo-controlled, double-blinded, phase III trial testing the hypothesis that Simvastatin (40 mg treatment started within 96 hours of the ictus, for 3 weeks) will reduce the incidence and duration of DINDs following subarachnoid hemorrhage. The target enrolment is 1,600 patients, with projected completion in February 2013.

Another study investigates the effect of statin therapy on cerebral blood flow in patients with aSAH, using a positron emission tomography [119]. A third study is randomizing 240 patients to 40 or 80 mg of simvastatin or placebo, with presence of DIDs listed as primary endpoint [120]. Finally, a double-blind, placebo, randomized, control trial with an estimated enrollment of 80 patients is being conducted in Sao Paulo with clinical outcome at 6 months being the primary endpoint [121].

## Conclusions

### Summary and interpretation

Several lines of evidence from both experimental and clinical studies suggests that statin use during sepsis, ALI/ARDS, and aSAH is beneficial. However, definitive conclusions cannot be drawn from the published evidence. Indeed, there is considerable heterogeneity of studies in type of statins, dosage, and duration of statin administration and case-mix. Moreover, many works are retrospective, providing less strong evidence than well-designed prospective studies. Waiting for the results of placebo-controlled, randomized trials, the intensive care use of statins should be cautious.

Indeed, the impact of statins side effects on large-scale utilization in intensive care is still not known. If serious side effects of statins, such as myopathy and rhabdomyolysis, are very rare in the general population (0.01% and 0.003% respectively) [122], the incidence and severity of these adverse effects could be higher in the critically ill. The lack of parenteral formulation of statins is another limitation, because the gastrointestinal absorption of statins might be impaired during critical illness. Furthermore, the pharmacokinetic profile could be altered during sepsis. Kruger et al. [123] demonstrated that when critically ill patients were given a single dose of 20 mg of atorvastatin, the plasma concentration peaked approximatively 18 times higher than those of healthy volunteers. Drage et al. [124] administered 40 mg of simvastatin to 27 critically ill patients, who also demonstrated higher plasma levels of both simvastatin and its active metabolite compared with healthy volunteers. Whether supratherapeutic plasma levels of statins in critically ill patients translates to an increased toxicity and requires adjustment remains unclear, especially when renal clearance might be impaired. The hepatic metabolization of statins might be altered as well, thereby increasing the risk of toxicity. Similarly, the metabolization of statins by the cytochrome P450 3A4 system may interfere with other medications commonly used in the ICU (i.e., amiodarone, macrolide antibiotics).

The right dosage is thus still undefined, as well as the right statins agent. Indeed, the type of statins used in cohort observational studies was very heterogeneous, raising the question of the similarity between the effects of the different agents. The majority of randomized, placebo-controlled trials plans to use simvastatin. As the other statins, simvastatin is metabolized by the liver, but its metabolite is cleared by the kidney and could accumulate in case of renal dysfunction, thereby increasing the risk of toxicity and in particular of clinical myopathy [125].

In sepsis, a major issue is the timing when statins should be started and stopped. An excessive inhibition of the inflammatory process could increase the susceptibility to secondary infections related to immune dysfunction [126-129]. It has been observed that more than 80% of nonsurviving patients die late after initial resuscitation in an immunosuppressive state, whereas patients who survive are those who spontaneously recover immune function [130]. Anti-inflammatory therapies may be harmful in septic patients [131,132]. This may be particularly true for statins that, above their anti-inflammatory effects, present suppressive effects on leucocytes. Indeed, monocyte deactivation is thought to be responsible for the impairment in antigen presentation and the decreased capacity to mount a proinflammatory reaction upon a secondary bacterial challenge [133]. As prophylaxis or in the very early management, the anti-inflammatory effects of statins may be protective in sepsis, but as the disease progress and multiorgan dysfunction becomes established, its side effects may prevail. Given these data, the objective of an ideal sepsis treatment should be to define the right action (i.e., blocking sustained inflammatory response, stimulate innate or adaptative immunity, restoring altered function) at the right time (early or delayed treatment) in the right patient (individualized therapy) [134].

## Abbreviations

HMG-CoA: 3-hydroxy-3-methylglutaryl coenzyme A; MAPK: Mitogen-activated protein kinase; PI3K: Phosphatidyinositol-3 kinase; NFK-B: Nuclear factor kappa B; MCP: Monocyte chemotactic protein; RANTES: Regulated upon activation normal T cell expressed and presumably secreted; VLA: Very late antigen; IL: Interleukin; TNF: Tumor necrosis factor; MHC: Major histocompatibility complex; TLR: Toll-like receptor; LFA: Lymphocyte function-associated antigen; iNOS: Inducible nitric oxide synthase; CRP: C-reactive protein; ICAM: Intercellular adhesion molecule; ELAM: Endothelial-leucocyte adhesion molecule; COX: Cyclooxygenase; PG: Prostaglandin; FiO_2_: Fraction of inspired oxygen; LIS: Lung injury score.

## Competing interests

The authors declare that they have no competing interests.

## Authors' contribution

IDL reviewed the literature, created the figures and tables, and drafted the manuscript. JCP revised the manuscript. All authors read and approved the final manuscript.
